# Multiloculated Liver Abscess Caused by Fusobacterium: Role of Karius Testing in Diagnosis

**DOI:** 10.7759/cureus.8823

**Published:** 2020-06-25

**Authors:** Hammad Zafar, Mamoon Ur Rashid, Bayarmaa Mandzhieva, Rima Shobar, Akriti G Jain

**Affiliations:** 1 Internal Medicine, AdventHealth Orlando, Orlando, USA

**Keywords:** pyogenic abscess, karius test, fusobacterium

## Abstract

Pyogenic liver abscess can be a major diagnostic and therapeutic challenge despite advances in cutting edge technologies. A patient presented with fever, right upper quadrant pain and diarrhea. CT revealed multiple hypodensities in both lobes of liver. The largest lesion was in the left lobe of liver and was multiloculated with thick septations. The causative organism was identified to be *Fusobacterium nucleatum* by Karius testing. The patient was discharged on six weeks of ertapenem therapy, which resulted in complete resolution. This is the first case of liver abscess where Karius testing was used to identify microorganism. It also highlights that multiloculated and difficult to drain liver abscesses caused by highly sensitive organisms can potentially be treated by intravenous antibiotics alone in immunocompetent patients.

## Introduction

Pyogenic liver abscess (PLA) can be a major diagnostic and therapeutic challenge, despite advances in cutting edge technologies. Development of new diagnostic methods, including CT and MRI, has made diagnosis more certain, and radiological intervention has changed spectrum of treatment. *Fusobacterium* is a rare cause of liver abscess that can affect immunocompetent hosts. We report a rare case of de novo hepatic abscess caused by *Fusobacterium* presenting as multiloculated abscess, diagnosed with Karius test and was managed nonoperatively with intravenous antibiotics. 

## Case presentation

**Figure 1:**
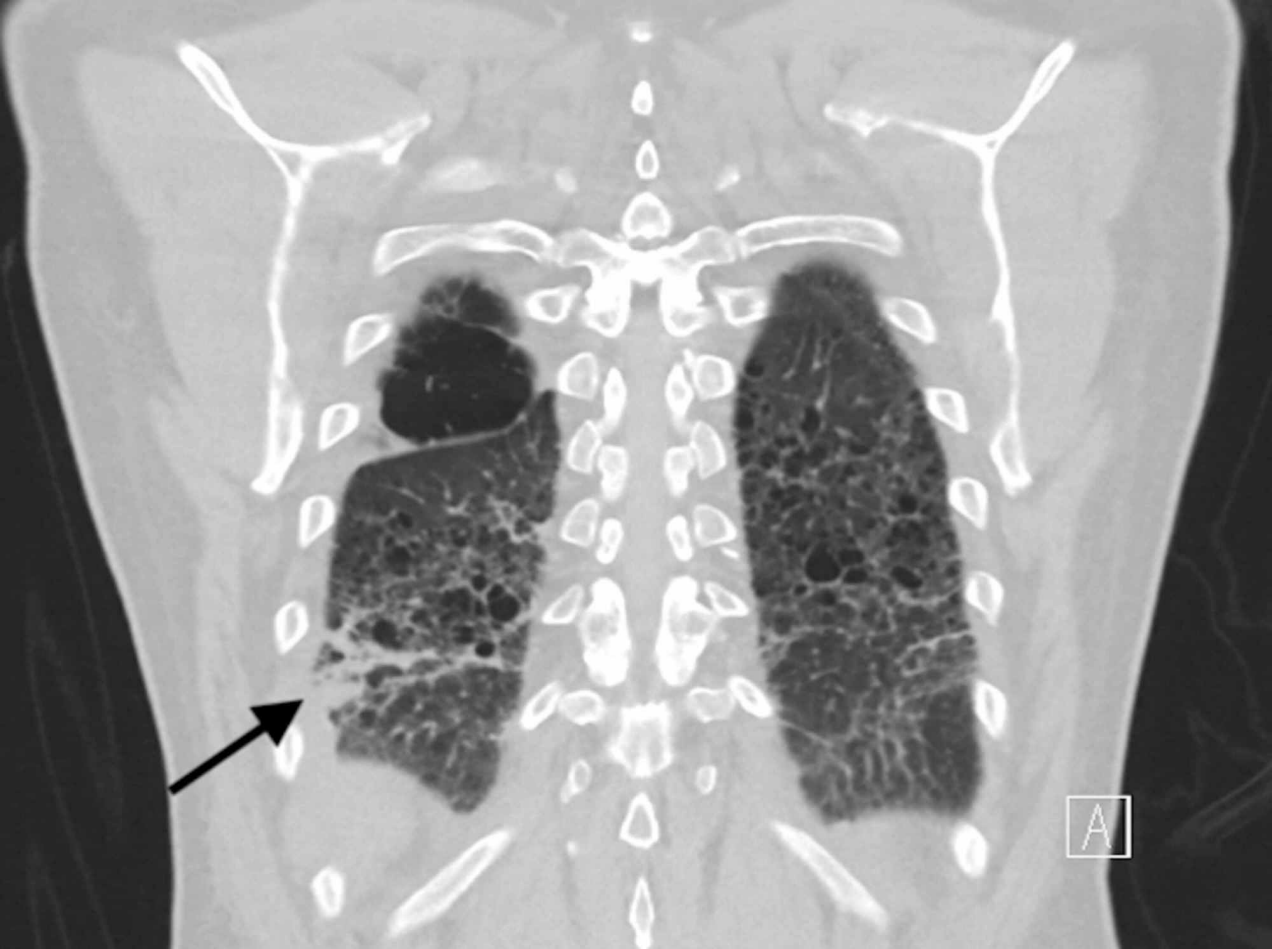
Emphysema and right lower lobe infiltrates on chest CT.

A 51-year-old male with a history of emphysema presented to an outside hospital with complaints of low-grade fever, flu-like symptoms, right-sided chest pain and dull right-sided abdominal pain accompanied by watery diarrhea for one week. Physical examination was positive for fever of 101°F, diaphoresis, right side basal crackles and right upper quadrant tenderness. Laboratory work was remarkable for leukocytosis of 19,000/μL with neutrophil predominance of 78%, total bilirubin of 2.4 mg/dl, aspartate aminotransferase (AST) 45 units/L, alanine aminotransferase (ALT) 36 units/L and alkaline phosphatase (ALP) 118 units/L. Chest x-ray was unremarkable, but CT chest (Figure 1) on the next day showed severe panlobular emphysema and focal consolidation in right lower lobe. Multiple hypodense lesions were seen in liver. 

**Figure 2:**
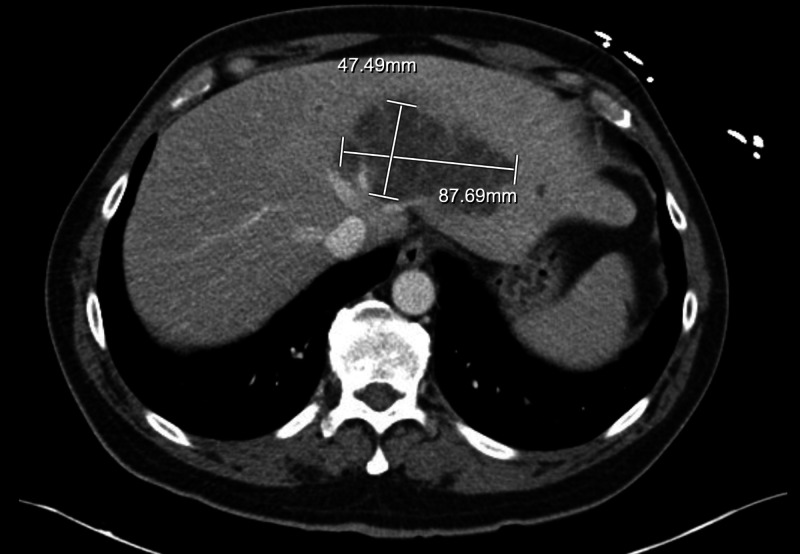
CT abdomen showing the largest multiloculated abscess (8 x 4 cm) in the left lobe of the liver.

Abdominal CT scan (Figure 2) was done, and it showed multiple lesions of liver concerning for metastatic disease. The patient was diagnosed with community-acquired pneumonia and was given one dose of piperacillin-tazobactam and was subsequently started on vancomycin and cefepime. MRI of the liver was ordered for further evaluation of hepatic lesions which showed hepatic lesions to be multiple hypointensities in both lobes of liver with largest measuring 8 x 4 cm in the left lobe of the liver. The largest lesion in the left lobe was multiloculated with thick septation.

**Figure 3:**
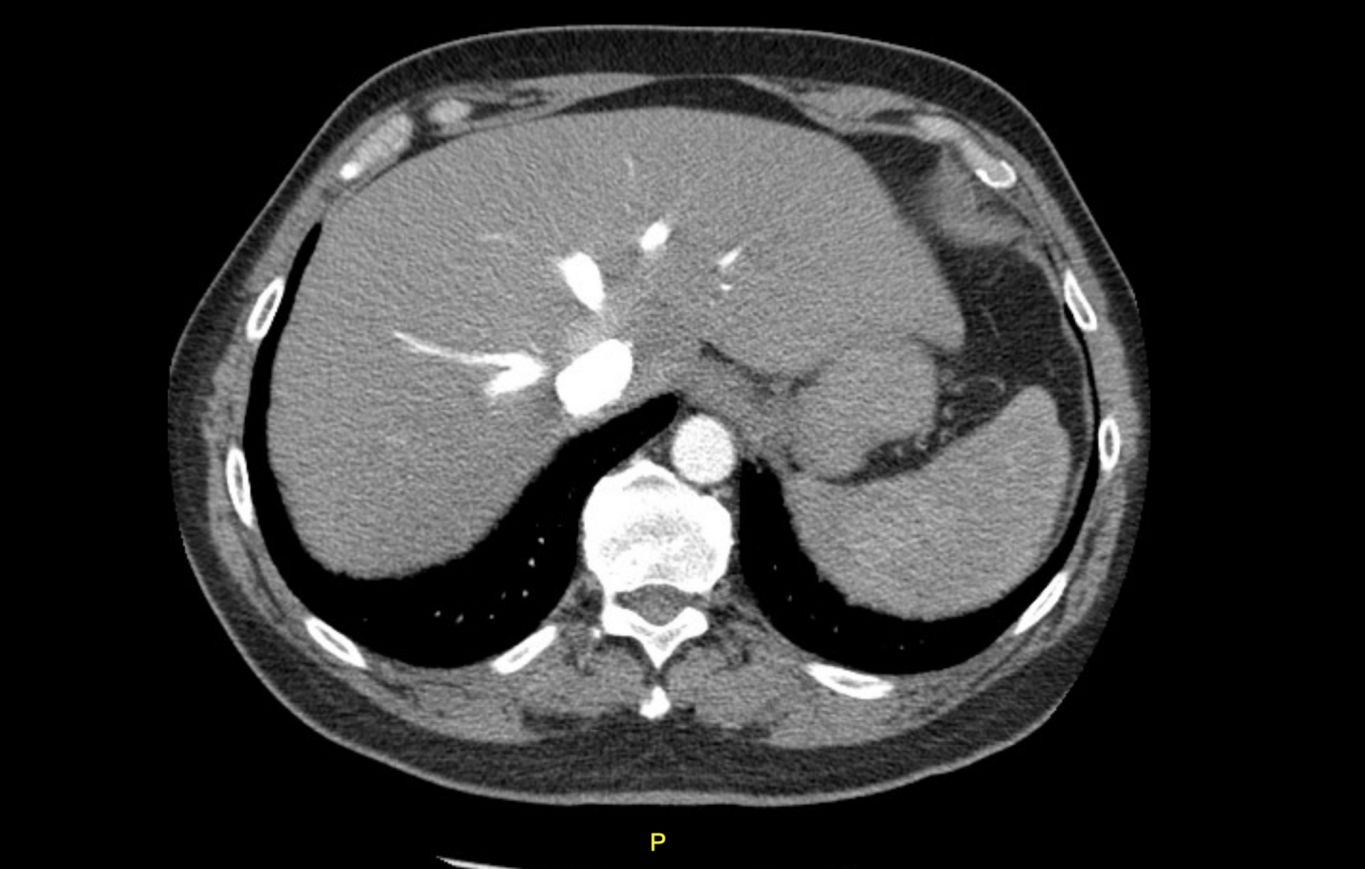
CT abdomen showing complete resolution of the abscesses after six weeks of treatment.

The patient had liver biopsy by interventional radiology, and pig tail catheter was placed with minimal drainage output. A colonoscopy was performed to identify source of infection, but it was unremarkable. The patient was transferred to tertiary care center for further evaluation from hepatology. No pathogen was identified from blood and fluid cultures. Broad-spectrum antibiotics were continued. Infectious disease workup, including *Entamoeba histolytica,* *Echinococcus*, *Toxoplasma*, *Giardia *and *Cryptosporidium*, remained negative. Karius testing was ordered to help identify the pathogen, and it was positive for *Fusobacterium nucleatum.* The patient denied any dental or oral problems, and subsequently underwent CT maxillofacial and neck to identify the source which was negative. No vegetations were seen on echocardiogram. Because of multiple septations, drainage of abscesses was not successful, and the patient was discharged on prolonged six-week course of ertapenem which led to complete resolution of the abscesses (Figure 3). 

## Discussion

The incidence of PLA is on the rise. Between 1994 and 2005, the incidence was 2.7-4.1 per 100,000 in the United States. The incidence is higher in Asian continent [1]. Liver abscess is potentially lethal disease with mortality rates reaching 100% without antibiotics. Mortality with proper antibiotic treatment still remains 2 %- 12 % [2]. Liver abscess usually presents subacutely with nonspecific signs and symptoms. Most common symptoms are fever, right upper quadrant pain, hepatomegaly and jaundice associated with decrease of appetite and weight loss [3].

It is more prevalent in males and most abscesses are located in the right lobe of the liver although multiple abscesses involving both lobes of the liver are not uncommon [2]. Causative microorganisms vary depending on local epidemiologic factors, but a shift in pathogens has corresponded with a recent rise in international travel. As a result, previously uncommon microorganisms should be considered in the differential diagnosis [2].

PLA is mostly biliary in origin, but hematogenous spread is also well established in literature [3]. The most common pathogens are streptococci, gram-negative bacteria, such as *Escherichia coli* and *Klebsiella,* and anaerobes [2]. *Fusobacteria* are peculiar amongst anaerobic organisms in their ability to cause perlious, clinically distinct, monomicrobial infections in different organs, including gastrointestinal tract and liver. Literature review in 2017 by Jayasimhan et al. identified 48 cases of liver abscesses caused by *Fusobacterium*. *F. necrophorum* and *F. nucleatum* were found as equal contributors. Hematogenous spread from periodontal disease was the most common attributable cause of *F. nucleatum* abscess followed by lower gastrointestinal tract disease. Lemierre disease has also been identified as likely source in some cases. Some cases are cryptogenic without identifiable source. Most of these cases occur in young or middle-aged individuals who are immunocompetent. They have no risk factors for liver disease including malignancy, end-stage renal disease or old age [4].

Ultrasound of abdomen has 40% sensitivity to diagnose liver abscesses, while abdominal CT scan is 90% sensitive [2]. Microbiological yield from blood and abscess culture is around 50%-60%. Early introduction of antibiotics without obtaining blood or fluid cultures could be a contributory factor. The Karius test is a blood test based on next-generation sequencing of microbial cell-free DNA. It can identify and quantify over 1,000 clinically relevant pathogens, including bacteria, DNA viruses, fungi and parasites. It is very useful to diagnose common/uncommon infections where conventional methods do not help identify causative organism [5]. Antimicrobials, interventional radiology or surgical drainage can be used, combined or as a single therapy. However, the combination of drainage with antibiotics has shown better outcomes in hospital stay, morbidity, fatality and complications [3]. Continuous catheter drainage is most commonly used technique for draining PLAs, while intermittent needle aspiration can also be used with comparable outcomes [6].

*Fusobacteria* are highly responsive to beta-lactam antibiotics. No resistance has been shown to amoxicillin in the last decade. Polymicrobial infection with mixed anaerobes is rare but if index of suspicion is high, metronidazole can be used in combination with beta-lactam with good clinical response. Prolonged intravenous antibiotic treatment might be warranted if the patient has risk factors for treatment failure, such as renal failure, hyperbilirubinemia, age > 65 years and Acute Physiology and Chronic Health Evaluation (APACHE) II score of greater than 154 [6].

A retrospective analysis done by Giangiuli et al. demonstrated higher 30-day admission rates in patients who were transitioned to oral antibiotics, mostly quinolones, at time of discharge as compared to IV antibiotics, mostly beta-lactams, in PLA [7]. Mohan et al. demonstrated sevenfold higher risk or colorectal cancer in patients with cryptogenic PLA. Screening colonoscopy may be warranted to rule out malignancy [8]. 

## Conclusions

Diagnosis and treatment of PLAs is difficult as illustrated by our case presentation. Presenting history, physical examination and laboratory markers can be highly variable. This case signifies the importance of newer diagnostic methods like Karius testing in microbiological diagnosis and nonoperative management of multiloculated liver abscess.
